# Oral mucosal disease: dilemmas and challenges in general dental practice

**DOI:** 10.1038/s41415-024-7080-x

**Published:** 2024-02-23

**Authors:** Philip A. Atkin, Rachel Cowie

**Affiliations:** 4141524575001 Consultant Oral Medicine and Honorary Senior Lecturer, Department of Oral Medicine, University Dental Hospital, Heath Park, Cardiff, CF14 4XW, UK; 4141524575002 Consultant Oral Medicine and Honorary Senior Lecturer, Department of Oral Medicine, Bristol Dental Hospital, Lower Maudlin Street, Bristol, BS1 2LY, UK

## Abstract

Oral medicine (OM) is a recognised component of all UK undergraduate dental programmes and practising dentists are expected to safely investigate and manage patients presenting with oral mucosal disease. Delivering OM care for patients in a general dental practice setting does however come with a number of challenges and dilemmas for practitioners.

General dental practitioners may be limited in their ability to arrange diagnostic tests such as biopsies or blood tests, important in reaching a definitive OM diagnosis. Lack of operator skill or lack of access to appropriate laboratory facilities to process diagnostic samples will likely contribute to this. In addition, general dental practitioners may feel underconfident to reliably interpret test results. Management of OM patients can also be time-consuming and may not generate a significant remunerative reward under current NHS payment systems.

OM is a subject that overlaps with several medical specialities, and up until 2010, required dual qualification in both undergraduate dentistry and medicine. Practitioners who have not undertaken OM training beyond undergraduate dentistry may lack confidence with the subject, and fear of misjudging a lesion of concern will certainly prompt referrals from primary care into hospital-based OM clinics.

## Key points

- Identifies expected knowledge, skills and standards of oral medicine care within primary care, as outlined by the General Dental Council and UK Committee of Postgraduate Dental Deans and Directors, and highlights the range of oral medicine conditions general dental practitioners may be faced with in practice.
- Outlines National Institute for Health and Care Excellence guidance regarding the urgent referral pathway for suspected head and neck cancer.
- Highlights factors limiting the practice of oral medicine in primary care and provides an opinion regarding potential changes that may improve access to oral medicine care and advice.

## Introduction

Oral medicine (OM) is one of a number of dental specialities that undergraduate dental students are exposed to during their training, with the expectation that on graduation they will be able to recognise oral mucosal disease, orofacial pain and other diagnoses, and either manage or refer appropriately. The General Dental Council (GDC) describes OM as ‘the speciality of dentistry concerned with the diagnosis and management of adult and child patients with chronic, recurrent and medically related disorders of the mouth, face and jaws. It interfaces between medicine and dentistry and specialists have enhanced training in the medical aspect of orofacial disease'.^1^

Graduating dentists leave dental schools with the knowledge, skills and experience dictated by the UK GDC curriculum as interpreted by their individual dental school in terms of didactic teaching and clinical experience.^2^ A search for ‘oral medicine' in the current undergraduate curriculum, *Preparing for practice*, finds no hits. In relation to mucosal disease, the GDC curriculum says the graduating dentist must:

- Recognise the early stages of mucosal abnormality and the importance of appropriate and timely referral (1.2.3)
- Recognise and manage acute dento-alveolar and mucosal infection (1.9.2)
- Recognise and manage acute dento-alveolar and mucosal trauma (1.9.3)
- Identify oral mucosal diseases and refer where appropriate (1.12.2).

On graduation, most new dentists will complete a year as a dental foundation trainee following the curriculum from the Committee of Postgraduate Dental Deans and Directors (COPDEND) UK.^3^ Again, there is no mention of ‘oral medicine' but references to ‘mucosal disease' are outlined below (Table 1).

**Table 1:** Extracts from COPDEND dental foundation training curriculum

Clinical domain 1: patient examination and diagnosis	7	Distinguish between mucosal, gingival and periodontal health and disease, and identify conditions which may require investigation, treatment or onward referral
	19	The clinical features associated with oral mucosal diseases, and identify conditions which may require treatment or onward referral (including urgent referrals for suspected head and neck cancer)
Clinical domain 8: non-surgical management of the hard and soft tissues of the head and neck	2	Understand and assist in the investigation, diagnosis and effective management of oral mucosal diseases, including the early referral of patients with possible pre-malignant or malignant lesions

## Oral mucosal disease and systemic disease

Over their career, dentists will inevitably encounter a significant number of patients suffering with oral mucosal disease, either related to mouth-limited conditions or as a manifestation of systemic disease. The mouth occupies an interesting site in the human body. The oral cavity mucosa transitions from the facial skin to the mucosa of the pharynx and gastrointestinal tract, and consequently, a number of dermatological conditions (for example, lichen planus, immunobullous disease) and gastrointestinal diseases (for example, Crohn's disease, ulcerative colitis and coeliac disease) will also present in the oral cavity. The oral mucosa will also display changes in relation to systemic mineral and vitamin deficiencies (for example, oral mucosal ulceration with iron deficiency), contact hypersensitivity (for example, lichenoid reactions) and inflammation secondary to mucosal irritation, for example, via trauma, heat or chemical products. Exposure to carcinogens, for example, alcohol or tobacco, may result in the development of intra-oral white patches, red patches (erythroplakia) or mixed lesions (erythroleukoplakia), all of which may have the potential to progress to dysplastic or malignant disease.

Medications taken for a wide variety of disease can commonly affect the oral cavity in a number of ways. Probably the most common side effect is reduced salivary flow, resulting in increased plaque accumulation and risk of dental caries, gingivitis and periodontal disease.^4^ Poorly controlled diabetes and raised blood sugar can reduce saliva flow further and produce an oral environment which encourages the growth of candida, causing additional problems in the mouth;^5^ respiratory disease and the frequent and regular use of inhalers can do the same.^6^ Patients taking systemic immunosuppressant drugs for management of chronic inflammatory disease (for example, rheumatoid arthritis), or following solid organ or bone-marrow transplant are also at increased risk of oral disease, including opportunistic candidal infections and mucosal ulceration.^7^

## Diagnosis and management of oral mucosal disease in general dental practice

From the GDC and COPDEND curriculum documents described earlier, there is an expectation that dentists will be able to diagnose, investigate and manage oral mucosal disease for their patient, or arrange onward referral to specialists in secondary care. Assessment of a patient's oral mucosa, as part of a thorough clinical dental examination, may reveal a number of manifestations of underlying systemic disease, including those outlined above. A good knowledge and understanding of medicine as it relates to dental care is therefore important, and delivered in the undergraduate curriculum as part of the teaching in human disease, as well as oral disease. Dental students will be taught how to examine the oral cavity, including thorough soft-tissue examination, and record their findings in an accurate and contemporaneous fashion. Approaches to oral cavity examination may differ subtly between individuals; however, consistency and maintenance of a systematic approach is key to ensuring examination is accurate and thorough. SDCEP has a number of clinician guides, among which is a useful assessment of oral mucosal tissue checklist and a scaled soft tissue diagram for recording any lesions found during the examination.^8^ Further information on how to conduct an oral cavity examination, including a video demonstration, can be found on the US National Institute for Dental and Craniofacial Research website^9^ and may be useful for some readers.

Dentists are acutely aware of the consequences of a missed or late diagnosis of oral cancer or potentially malignant disease, and the National Institute for Health and Care Excellence (NICE) guidance relating to this advises use of a suspected cancer pathway referral (for an appointment within two weeks) in people with either:^10^

- Unexplained ulceration in the oral cavity lasting for more than three weeks, or
- A persistent and unexplained lump in the neck, or
- A lump on the lip or in the oral cavity, or
- A red or red and white patch in the oral cavity consistent with erythroplakia or erythroleukoplakia.

From their own clinical experience, and from reading other papers in this special issue, dentists and other dental care professionals will be aware however that there are numerous conditions which present in the oral cavity as red or white lesions, mixed red and white lesions, or ulcers and lumps which can be entirely benign but necessitate referral given patient symptoms or diagnostic uncertainty, particularly in lesions that mimic the appearance of potentially more sinister conditions or even frank malignancy. Clinicians may consider an urgent suspected cancer (USC) referral according to NICE guidelines (Table 2).^10^

**Table 2:** Common and systemic disease-associated oral mucosal lesions with referral urgency. (In patients that use tobacco or take alcohol above national guidance, a more urgent referral than indicated may be appropriate)

Lesion type	Urgency of referral
**Ulcers**	
Non-healing ulcer lasting more than three weeks*	USC
Trauma-induced oral ulceration, non-healing despite addressing local cause	USC
Minor recurrent oral ulceration	Non-urgent
Major recurrent oral ulceration	Non-urgent
Oral ulcerations associated with eg Crohn's disease, Behcet's disease, anaemias	Non-urgent
**Red/white lesions**	
Persistent red patch (erythroplakia)*	USC
Persistent red and white patch (erythroleukoplakia)*	USC
Bilaterally symmetrical red, red/white or white patch (possible lichen planus)	Urgent (but not USC)
Unilateral red or red/white lesion immediately adjacent to a dental restoration (eg amalgam, gold crown) - possible lichenoid reaction	Urgent (but not USC)
Isolated white patch, thickened and well defined adjacent to a sharp or broken tooth, denture or orthodontic appliance - possible frictional keratosis	Non-urgent
Mixed red/white lesions associated with eg systemic lupus erythematosus	Non-urgent
**Lumps and bumps**	
A lump on the lip (especially a sun-exposed site) that has features of malignancy*	USC
Oral mucosal pink, soft, fleshy lesion in a site of common trauma (eg fibrous polyp)	Non-urgent
Oral mucosal (especially lower lip) fluid-filled soft fluctuant lesion (eg mucocoele)	Non-urgent
Gingival pink/red soft tissue lump, friable and bleeding and present for more than four weeks in a pregnant patient (eg pyogenic granuloma, pregnancy epulis)	Non-urgent
Multiple mucosal fibrous polyps associated with eg neurofibromatosis	Non-urgent
Key: * = As described in NICE guidance for head and neck cancer referral^11^	

## Referral patterns of primary care GDPs to OM (and OMFS) secondary care services

The majority of patients seen in hospital-based OM out-patient clinics come via referral from a general dental practitioner (GDP). Smaller numbers of patients are seen following referral by general medical practitioners (GMPs) and secondary care services.^11^ A 2019 paper by Friesen and colleagues looked at the referral patterns of GDPs to a Canadian university OM clinic.^12^ Of 924 patients examined, 752 (81.4%) were referred from GDPs. The most common type of lesion to be referred was a white/red lesion (n = 351; 38%), the most common site for referred patients was the tongue (n = 292; 31.6%) and the most common diagnosis was ‘immune-mediated', including lichen planus (n = 265; 28.7%). A 2021 paper from Coppola and colleagues looked at similar referral data for a university OM centre in Southern Italy, finding that of 583 patient first visits, 62.9% (n = 367) were referred by GDPs. The most common diagnosis was ‘immune-mediated disease', including lichen planus and immunobullous disease.^11^ Referrals are seemingly commonly for the purpose of either exclusion of dysplastic or malignant disease or to address the patient's distress with symptoms from, or appearance of, a benign lesion, that is, a fibroepithelial polyp or squamous papilloma. A UK 2006 paper looking back 30 years at over 44,000 specimens submitted for histopathological analysis revealed the four most common diagnoses following oral mucosal biopsy as fibrous hyperplasia (n = 6,458), lichen planus (n = 2,973), hyperkeratosis (n = 2,481) and epithelial dysplasia (n = 1,280).^13^

In the UK, where OM specialist services are located within a few university dental hospitals and schools, many GDPs will refer patients to their local oral and maxillofacial surgery (OMFS) outpatient clinic for diagnosis and treatment. We know that there is a significant amount of limited scope OM activity performed by oral surgeons and OMFS colleagues in such settings, with up to 40% of patients attending these clinics because of conditions which would fall within the remit of OM, including significant amounts of oral mucosal disease.^14^

## Challenges of practising OM in primary care

**Fig. 1:**
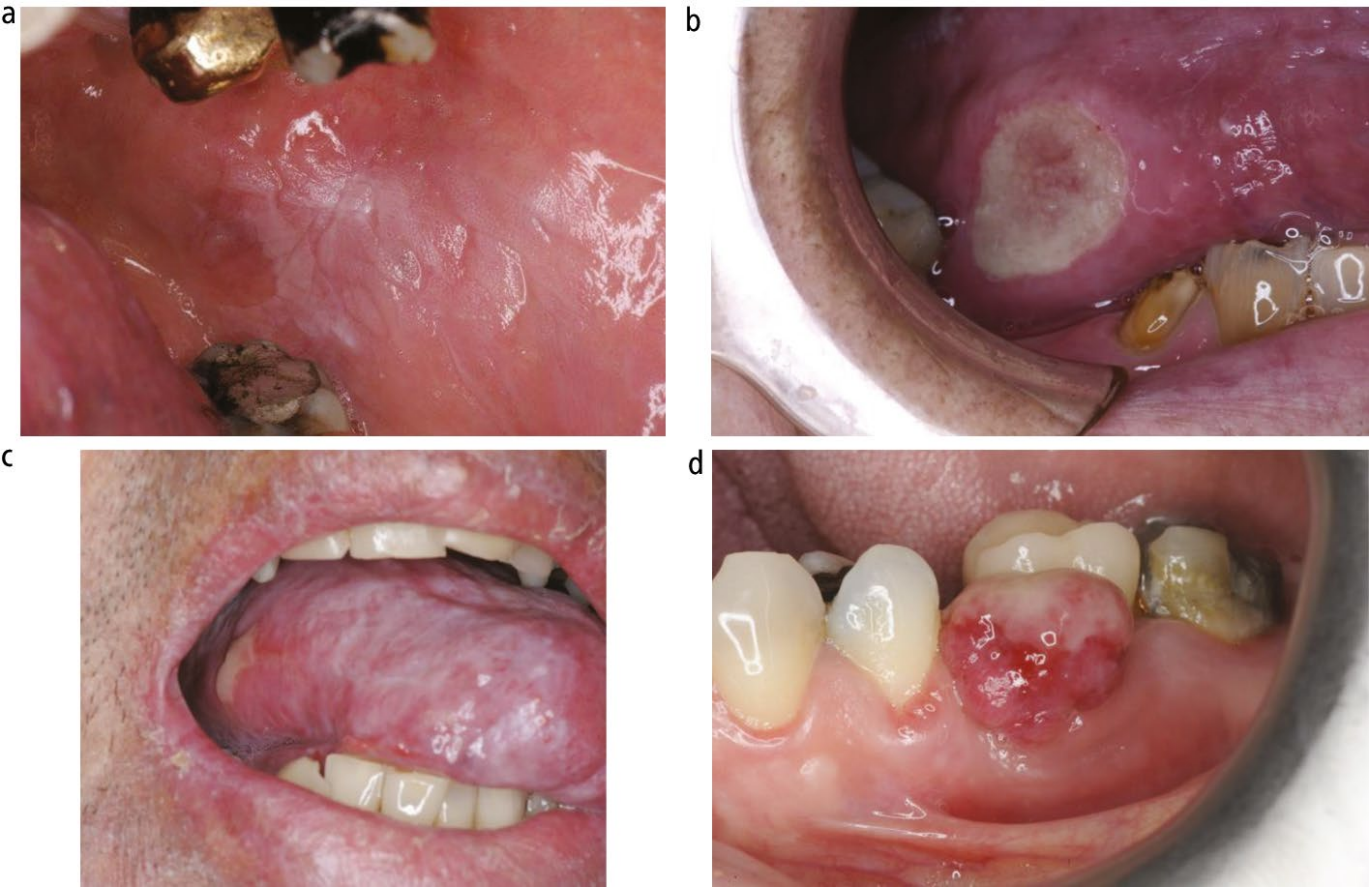
a, b, c, d) Examples of GDP-referred oral mucosal lesions

Figure 1 shows examples of soft tissue lesions in patients referred from their family dentist to OM clinics for diagnosis and treatment. Which, if any, of the lesions are malignant? (Table 3)

**Table 3:** Diagnoses and additional information for soft tissue lesions in Figure 1

Image	Diagnosis	Additional information
Panel A	Lichenoid reaction to dental material	Close approximation to dental amalgam (lower molar) and gold crown (upper molar). Patch-testing to confirm
Panel B	Traumatic ulcer	Close approximation to sharp and broken teeth
		Tooth smoothing/extraction and review of healing ulcer site
Panel C	Graft-versus-host disease	Recent bone-marrow transplant and patient is now taking systemic immunosuppressive drugs
Panel D	Pyogenic granuloma	Local irritation at site of lesion (eg calculus, overhanging amalgam). Excision biopsy and histopathology confirmation of diagnosis

To establish a clinical diagnosis and definitively reach a conclusion regarding whether or not any the presentations above show dysplastic or malignant disease, full assessment/disclosure of the patient's medical history, review of systems and diagnostic histopathology/serology would need to be considered. Although none of the clinical images actually show malignant lesions, the appearance of any and all lesions shown in these clinical images could be consistent with malignancy and therefore, such cases must be treated as such until proven otherwise. To do this, a combination of experienced assessment, a sound understanding of related medical disease and the access and skills to perform and interpret diagnostic tests, such as histopathology, is essential.

Presentation of such lesions in primary care settings may understandably therefore cause many practical and diagnostic challenges for a GDP.

### Limited access to diagnostic tools and financial barriers

While some patients presenting with oral mucosal lesions may receive a diagnosis based purely on the clinical appearance and history of a lesion, more commonly, OM specialists (who are almost exclusively based within university dental hospital departments) will want a series of further investigations, including blood tests and biopsies, to provide definitive diagnoses before then going on to manage the lesion or disease.

Hospital clinics, unlike most general dental practices, are usually well-equipped with diagnostic equipment, readily enabling microbial sampling, blood tests and biopsies. Similarly, well-established pathology and microbiology laboratory services, needed to process any diagnostic samples, are almost always available and accessible to hospital clinicians. This is often not the case in primary care.

The practice of OM outside of a hospital setting is a challenging fit in the current NHS remuneration system. The relative length and complexity of OM treatment plans means that there is often little remunerative reward compared to treatment plans heavily geared towards operative treatment. The difficulty of measuring and remunerating such OM care is certainly an additional barrier to the provision of optimal patient management in the practice setting.

Comprehensive patient assessment is often required for the diagnosis and management of more complex oral mucosal diseases, in particular those presenting alongside systemic symptoms. The time implications that such assessment has for a practitioner already managing high-volume patient numbers with back-to-back appointments should not be underestimated. Arranging diagnostic or monitoring blood tests from a dental practice in liaison with the patient's GMP or local hospital and following-up of results is time-consuming and attracts no NHS fee.

### Scope of practice

While OM is a component of all UK undergraduate dental programmes, the curriculum space available for this will vary considerably and some dental students may have limited exposure to OM teaching before they graduate. Even if undergraduate education provides a student with a significant exposure to OM patients before degree completion, it is to be expected that this knowledge will almost certainly fade with time, given that it will rarely be applied regularly within the scope of general dental practice.

General dentists may often not feel confident to reliably perform relevant diagnostic tests, for example, mucosal biopsy or blood tests, nor may insurance/indemnity policies cover the performance of such procedures. Furthermore, when investigations are undertaken, the dentist would need to be able to interpret tests results considering the mucosal disease in question, or medications being monitored, and this would be felt to be beyond the scope of practice for the majority of practitioners.

Many oral mucosal conditions are chronic diseases requiring ongoing management with topical or systemic medications, with drugs and doses being altered to match changes in disease activity. Once a diagnosis is reached, the GDP has only a very limited range of medications available to them on the Dental Practitioners' Formulary (DPF)^15^to manage their patient and their mucosal disease. Dentists may also have only very limited experience with the prescription of such medicines.

### Patient expectations and fear of litigation

OM diagnoses are reached following comprehensive dental and medical assessment of a patient, often involving questioning regarding extra-oral or systemic symptoms. Patients may be reluctant to divulge medical details to dentists, who they may judge as unqualified to manage conditions traditionally dealt with by medical doctors. Consequently, a dentist may be inadequately supplied with key information essential to establishing a definitive diagnosis. Dentists may also feel underqualified to adequately answer questions regarding details of patients' conditions, in particular the complexities of any associated systemic illness, and would therefore be more likely to refer the patient into a specialist setting, where this information will be more readily and reliably given to the patient.

Fear of missing or misjudging a worrying lesion with resulting potential litigation clearly prompts numerous referrals to hospital OM clinics each year.^16^^,^^17^ As a result, a large proportion of cases referred urgently or under two-week wait guidance are made for benign lesions that could have been referred safely along routine pathways. Similarly, this is the case for specialities allied to OM, for example, OMFS and ear, nose and throat surgeons, who also accept referrals for symptoms/lesions suspicious of head and neck cancer. A 2019 Glasgow-based study published data regarding outcomes of 2,116 urgent head and neck cancer referrals via the urgent suspicion of cancer pathway over a one-year period. Cancer identification rates through this route were low, with only 152 (7.6%) cases resulting in a primary head and neck cancer diagnosis.^18^

## Discussion

In consideration of all of the above, it is evident that the GDC and COPDEND have expectations in their curriculums that GDPs will diagnose and manage the oral mucosal lesions of their patients. In some cases, this may be quite straightforward, for example, prescribing topical antifungal medication for a candidal infection, salivary substitutes for xerostomia, or topical corticosteroid formulations, that is, soluble betamethasone mouthwash, for a patient with oral lichen planus and associated mild symptoms. Other oral mucosal lesions will, however, require biopsy for definitive diagnosis or to exclude malignancy, and certain disease management and ongoing care may require drugs not available on the DPF. NHS dental practice may also not be the ideal setting to discuss complex investigation, diagnosis and management options, for which there is no matching remuneration scale in a fee-per-item style NHS dental contract and limited clinical time in which to provide this service.

Increasingly, dental practices are using digital or electronic referral systems which provide for the inclusion of a digital image (in the case of OM, a clinical photograph) which, if of sufficient quality, can be reviewed by an OM specialist and a diagnosis and treatment plan suggested, avoiding the need for a consultation in secondary care.^19^ Non-healing ulcers or mixed red and white lesions will likely need biopsy for definitive diagnosis, so a referral can be expedited by the receiving specialist on the basis of a good clinical image. Inclusion of such an image within a referral should be encouraged, therefore, as it allows for more accurate and efficient triaging of patients. It should also provide reassurance for the referring GDP that their referral will be triaged appropriately, irrespective of whether it is sent as routine or urgent.

Use of videoconferencing technologies to diagnose and provide advice about treatment is an approach that was used extensively during the recent COVID-19 pandemic. While not without challenges, this technology, and in particular telemonitoring of patients initially assessed and diagnosed on a face-to-face basis, continues to have a place in OM patient care and may offer particular benefit for patients living in rural and remote areas who face longer travel distances to hospital sites.^20^^,^^21^^,^^22^

The current NHS Advice and Guidance system^23^ provides an interface between primary and secondary care services and allows GMPs access to consultant advice via synchronous (for example, telephone calls) or asynchronous (for example, email) means. This enables GMPs to seek specialist advice on issues such as patient tests results, treatment planning advice or even appropriateness of onwards referral into secondary care. This system facilitates shared decision-making between primary and secondary care and reduces the amount of unnecessary referrals sent into specialist hospital centres. Extension of this service to allow more widespread use within primary care dentistry may help overcome some of the previously outlined challenges faced by GDPs looking to engage with OM practice for their patients.

## Conclusion

In conclusion, some dentists may have a good working knowledge of local and systemic disease relating to the oral mucosa but are limited in their ability to investigate (for example, blood tests, biopsy), prescribe and monitor both the mucosal disease itself or the effects of systemic drug treatments necessary to manage such disease. There is a large discrepancy between what primary care dentists are able to do for their patients and what dental hospital-based OM specialists can do. Postgraduate courses aimed at developing the knowledge and skills of interested GDPs may produce groups of clinicians with the ability to contribute to a formal managed clinical network (MCN). Programmes specifically developed to produce dentists with enhanced skills, also known as Level 2/Tier 2 practitioners, from royal colleges^24^ or postgraduate dental deaneries^25^are in development, including for OM. MCNs might go some way to addressing providing OM care outside of a dental hospital environment,^26^ but dentists with enhanced skills working within an MCN will need to be based in regional district general hospitals or non-acute hospitals in order to have access hospital laboratory services for biopsy and blood tests, and also to facilitate prescription of medication beyond the limited number of drugs available in the DPF. There is much potential for progress in this field; however, there must be sufficient organisation and funding of appropriate systems and pathways if effective change is to be possible.
